# Surfactant replacement therapy as promising treatment for COVID-19: an updated narrative review

**DOI:** 10.1042/BSR20230504

**Published:** 2023-08-09

**Authors:** Khaled Khudadah, Alaa Ramadan, Ahmed Othman, Neveen Refaey, Amr Elrosasy, Ayoub Rezkallah, Toka Heseba, Mostafa Hossam El Din Moawad, Ammar Mektebi, Sewar A Elejla, Mohamed Abouzid, Basel Abdelazeem

**Affiliations:** 1 Kuwait Oil Company Ahmadi Hospital, Al Ahmadi, Kuwait; 2 Faculty of Medicine, South Valley University, Qena, Egypt; 3 Women’s Health department, Faculty of Physical Therapy, Cairo University, Cairo, Egypt; 4 Faculty of Medicine, Cairo University, Cairo, Egypt; 5 Faculty of Medicine, University of Algeirs, Algeirs, Algeria; 6 Department of Hematology Laboratory and Blood Transfusion, Hospital Center University Lamine Debaghine, Algeirs, Algeria; 7 Faculty of Medicine, Assuit University, Assuit, Egypt; 8 Faculty of Pharmacy, Clinical Department, Alexandria University, Egypt; 9 Faculty of Medicine, Suez Canal University, Ismailia, Egypt; 10 Faculty of Medicine, Kutahya Health Sciences University, Kutahya, Turkey; 11 Faculty of Medicine, Alquds University, Jerusalem, Palestine; 12 Department of Physical Pharmacy and Pharmacokinetics, Faculty of Pharmacy, Poznan University of Medical Sciences, Rokietnicka 3 St., 60-806 Poznan, Poland; 13 Doctoral School, Poznan University of Medical Sciences, 60-812 Poznan, Poland; 14 McLaren Health Care, Flint, Michigan, U.S.A.; 15 Michigan State University, East Lansing, Michigan, U.S.A.

**Keywords:** Acute respiratory distress syndrome, Alveolar cells type 2, COVID-19, pulmonary surfactant, SARS-CoV-2

## Abstract

Patients with COVID-19 exhibit similar symptoms to neonatal respiratory distress syndrome. SARS-CoV-2 spike protein has been shown to target alveolar type 2 lung cells which synthesize and secrete endogenous surfactants leading to acute respiratory distress syndrome in some patients. This was proven by post-mortem histopathological findings revealing desquamated alveolar type 2 cells. Surfactant use in patients with COVID-19 respiratory distress syndrome results in marked improvement in respiratory parameters but not mortality which needs further clinical trials comparing surfactant formulas and modes of administration to decrease the mortality. In addition, surfactants could be a promising vehicle for specific drug delivery as a liposomal carrier, which requires more and more challenging efforts. In this review, we highlight the current reviews and two clinical trials on exogenous surfactant therapy in COVID-19-associated respiratory distress in adults, and how surfactant could be a promising drug to help fight the COVID-19 infection.

## Introduction

**Figure 1:**
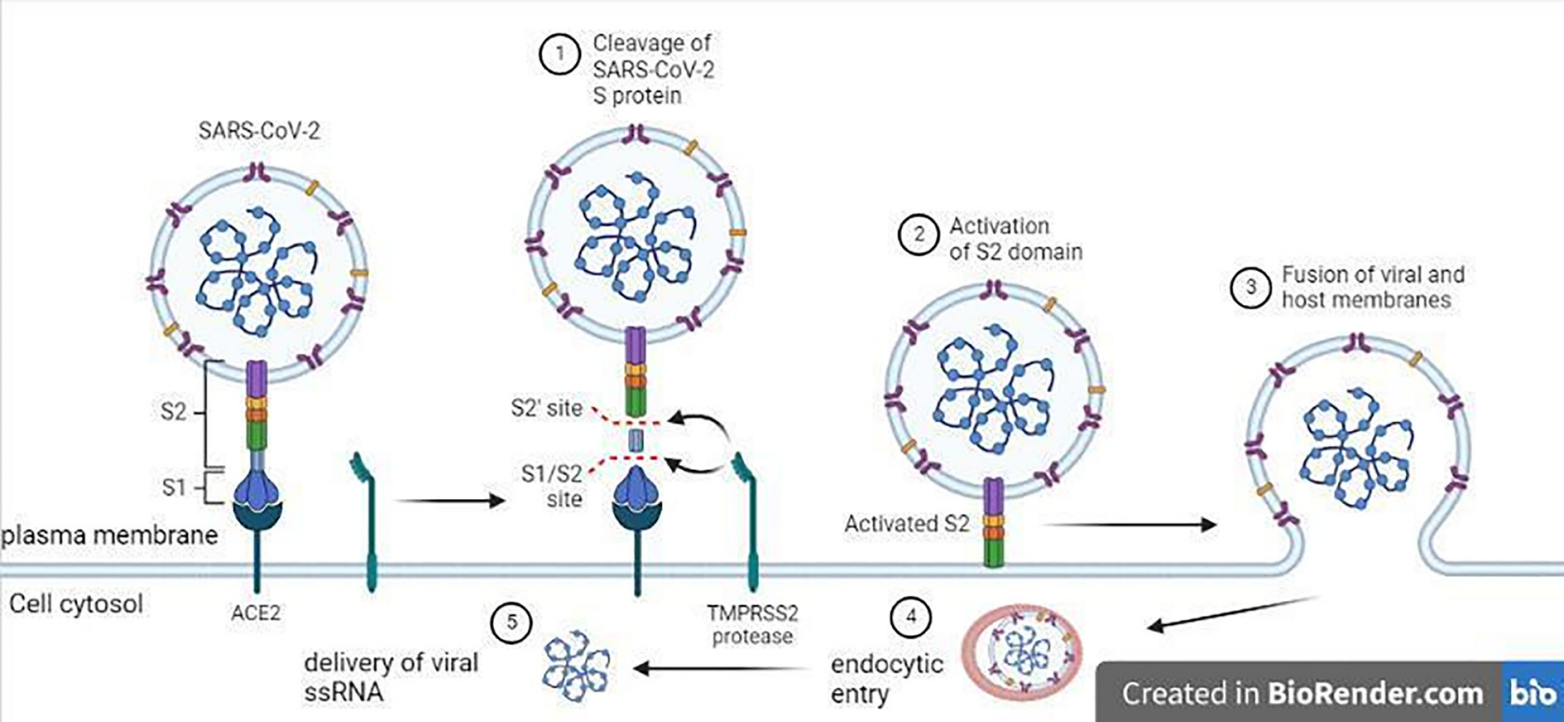
Structure of SARS-CoV-2 and methods for entrance into host cells The nucleocapsid, membrane, envelope, and spike protein are the four structural proteins that are encoded by the single-stranded RNA viral genome. Both options for viral internalization—via direct fusion of the viral envelope and the host cell plasma membrane or—via endocytic entry, followed by fusion between the viral envelope and the endosomal membrane—are triggered by the binding of Si of the viral spike protein to the ACE-2 receptor on host cells. Abbreviations: ACE-2, angiotensin-converting enzyme 2; SARS-CoV-2, severe acute respiratory syndrome coronavirus-Z; TMPRSS2, transmembrane protease serine 2, subunits 1, 2, and 2. From ‘Mechanisms of SARS-CoV-2 Viral Entry,’ modified, by BioRender.com (2021). Retrieved from https://app.biorender.com/biorender-templates.

COVID-19 is an encapsulated, single-stranded, positive-sense RNA virus that can cause serious illnesses due to its broad-ranging tropism [1,2]. Severe COVID-19 represents viral pneumonia caused by SARS-CoV-2 infection, which results in acute respiratory distress syndrome (ARDS) [3]. ARDS is characterized by lung inflammation and pulmonary edema. SARS-CoV-2 virus mainly attacks alveolar cells type 2, whereupon it induces apoptosis, cell damage, and decreased pulmonary surfactant synthesis [4] (Figure 1). Angiotensin-converting enzyme 2 (ACE2) is recognized as playing a significant role in host–virus interaction. However, new gene-ontology investigations have revealed that alveolar type 2 (AT2) cells represent 83% of lung cells expressing the ACE2 gene, and the progressive damage of AT2 limits surfactant production [5]. Pathogens are thought to be opsonized by pulmonary surfactant, which makes it easier for cells of the innate immune system to phagocytose them [6]. It may be most beneficial if preventative measures and a combination of medicines are available for people who are already infected. Numerous existing medications, such as anti-inflammatory agents, have been evaluated in response to the pressing need to stop the COVID-19 epidemic [7].

There are currently just a few trials that describe the possibility of using exogenous surfactants as adjuvant therapy for COVID-19-associated ARDS in a limited number of patients [8,9]. Using exogenous surfactant improved static lung compliance and oxygenation and had the propensity to lower mortality rates. This study will briefly discuss the role of surfactants and the effectiveness of exogenous surfactant therapy in treating neonatal and adult respiratory distress syndrome. In addition, we aim to review the planning and analysis of clinical trials on exogenous surfactant therapy for COVID-19.

## SARS-CoV-2 mechanism

**Figure 2:**
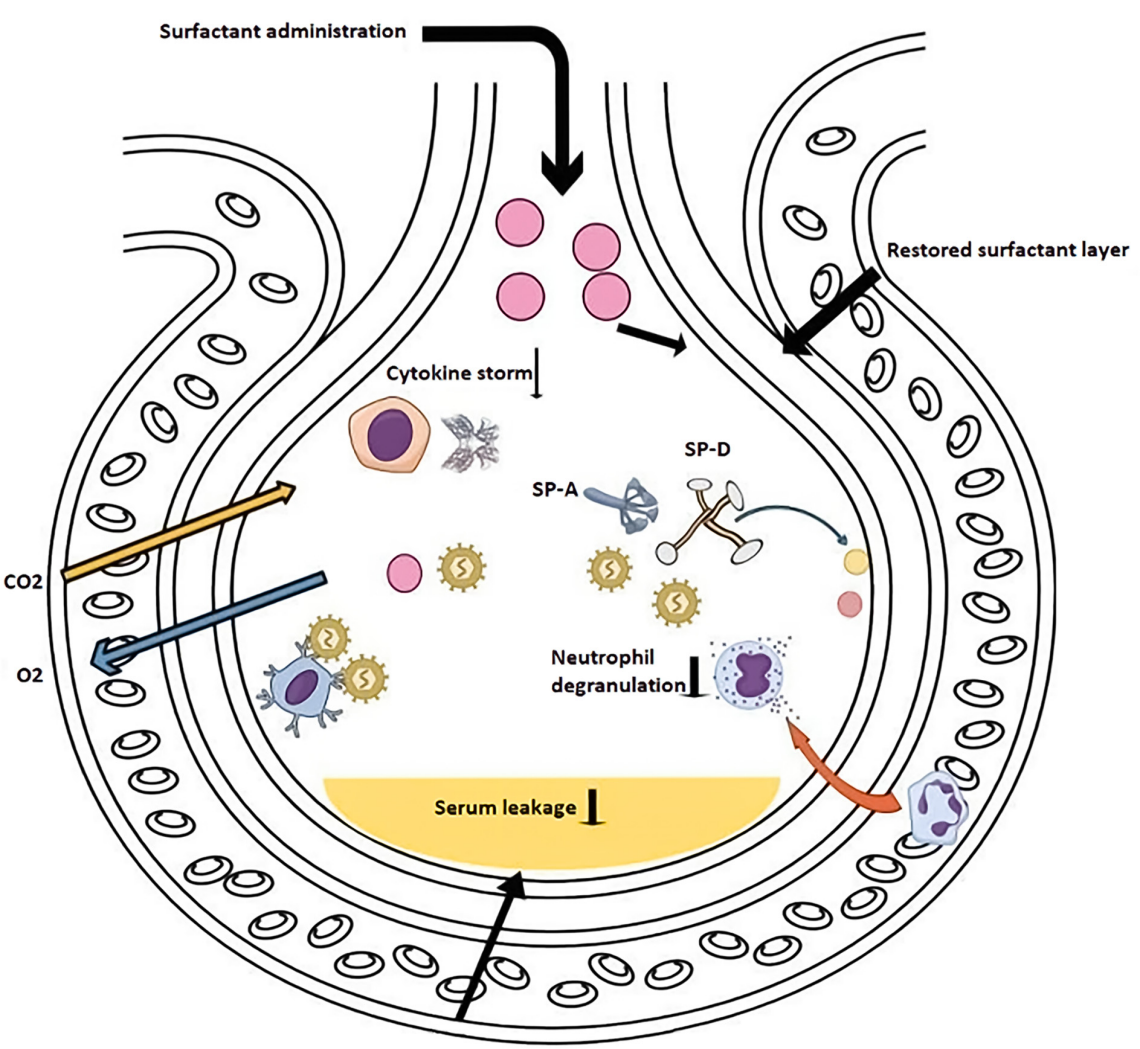
COVID-19-related acute respiratory distress syndrome: 1-Cytokine storm 2- neutrophil degranulation and serum leak 3- pneumocyte destruction Retrieved from https://app.biorender.com/biorender-templates.

SARS-CoV-2 is thought to target alveolar type-II cells, the lung cells in charge of producing surfactant, similar to SARS-CoV. Inflamed cells emit endogenous chemicals that trigger Toll-like receptor activation, inflammatory mediators, and inflammation synthesis. Along with the surfactant’s continued decline brought on by the demise of these type II pneumocytes, these effects (and possibly the increased activity of secretory phospholipase A2 [sPLA2]) [10,11] promote pulmonary edema, a hallmark of COVID-19. Pulmonary edema, in turn, leads to ARDS (Figure 2).

Early on, the pulmonary failure brought on by COVID-19 appears to be very different from other forms of ARDS [12,13]. For instance, many COVID-19 patients first exhibit hypoxemia with sustained lung compliance, referred to as the L-type presentation provided by phosphatidylcholine. L-type patients frequently change into the second or H-type clinical presentation, nevertheless. Only after significant phosphatidylcholine depletion and mass destruction of AT2 cells, which show high elastance and low compliance, will the surfactant’s ability to reduce surface tension be lost [13,14]. Consequently, only the H-type reproduces the lung characteristics of premature children with low surfactant production.

**Figure 3:**
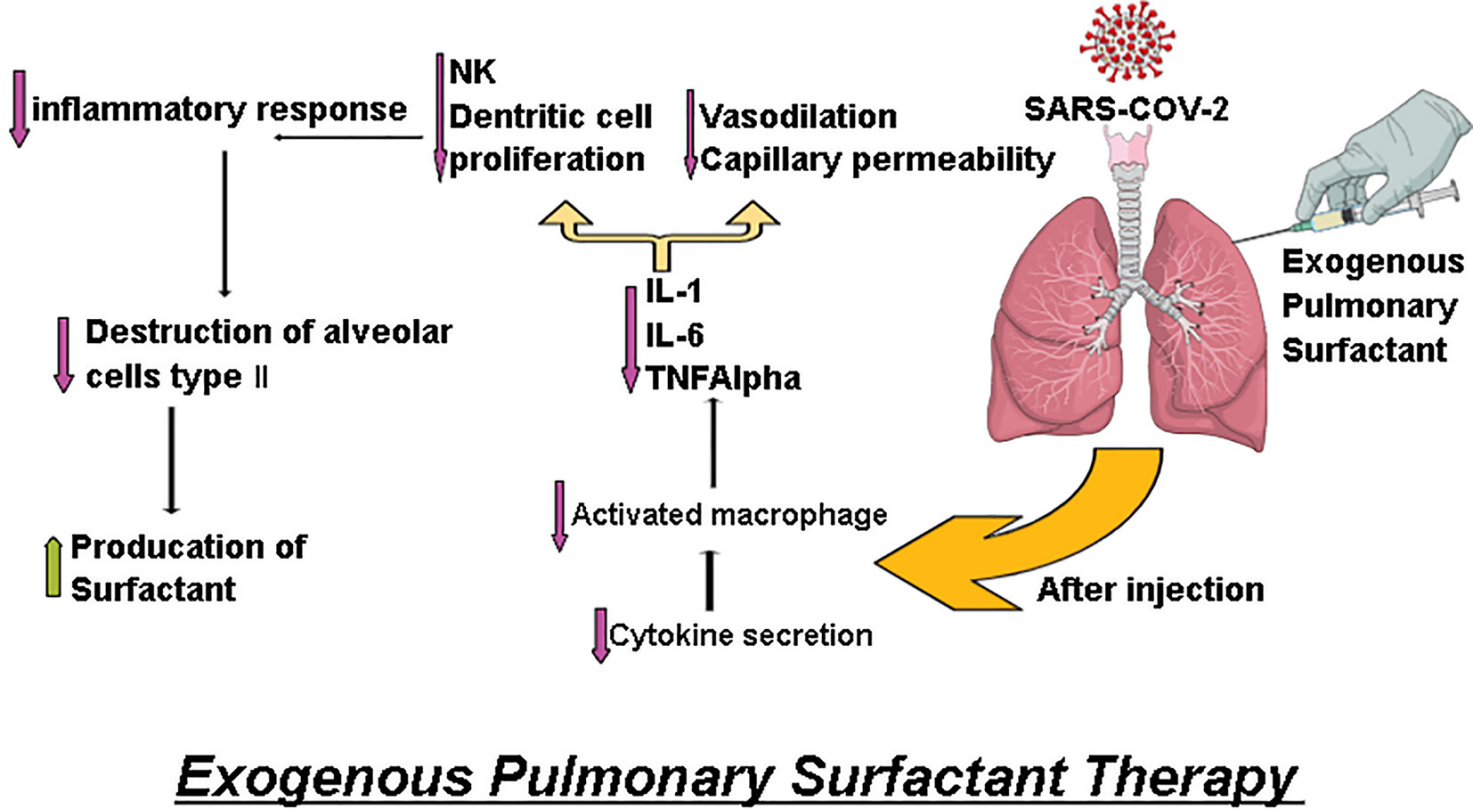
Effect of exogenous pulmonary surfactant therapy

Exogenous surfactant administered via the lungs is predicted to counteract this chain of events in a variety of ways (Figure 3), including (i) restoring surfactant levels to protect against increased surface tension in the lung, (ii) limiting the production of damage-associated molecular patterns (DAMPs) from activating the innate immune system to minimize inflammation and inflammatory damage by suppressing the activation of toll-like receptors [15], and (iii) reducing pulmonary edema through the combination of the first two actions.

Finally, excessive inflammation is linked to the hypercoagulopathy that is occasionally observed in COVID-19 individuals (e.g., notably elevated C-reactive protein and D-dimer levels). As a result, phosphatidylglycerol’s capacity to block PAMP- and DAMP-induced sequelae may likewise lessen these COVID-19 effects.

## Structure and uses of surfactant

The most prevalent surfactant component is lipids, as it contains a high amount of dipalmitoylphosphatidylcholine (DPPC), the most abundant phospholipid in surfactant and the most important in reducing surface tension, and phosphatidylglycerol that indicates lung maturity [16–18]. The surfactant also contains two small hydrophobic proteins, surfactant proteins (SP)-C and SP-B, which enable the lipids to adsorb and spread rapidly into the air-liquid interface. Without these proteins, lipids do not move out from their assembly origin into the interface [19–21]. The other two oligomeric proteins, SP-A and SP-D, have immunomodulatory properties by binding to innate immunity receptors and helping in cytokine production, which are hydrophilic [21,22]. DPPC allows the surfactant to reach maximal packing due to its saturated acyl chains, which can have an entirely straight conformation as opposed to other unsaturated acyl chains. Phosphatidylinositol and phosphatidylglycerol are the most prevalent anionic phospholipids in surfactants. They are considered cofactors that form selective interactions with the hydrophobic cationic groups of SP-C and SP-B to catalyze their specific function. Cholesterol amounts are highly variable, but a high percentage of cholesterol may indicate pathological conditions such as ARDS [23,24]. However, it is still unclear whether high cholesterol is a consequence or a cause of a maleficent surfactant system. Recently, it has been thought that limited amounts of cholesterol are still important for unique interactions with hydrophobic proteins [25,26]. The basic compositional structure of surfactant requires saturated/unsaturated proportions of anionic phospholipids, DPPC, hydrophobic proteins, and cholesterol. Pathological conditions leading to a defect in these components worsen surfactants’ performance.

The surfactant can be extracted by bronchoalveolar lavage in animals, and the whole material coating both upper and distal airways can be collected. Suppose extraction is done by bronchoscopy in patients. In that case, the material obtained will be just from the area washed, the cells of the material are removed, and the rest is centrifuged to yield the surfactant, which consists of 80% phospholipids, 10% proteins such as SP-C and SP-B, and 10% neutral lipids [27]. Calfactant, from calf lung, contains normal amounts of cholesterol and works well in surfactant replacement therapy of premature babies [28–30]. Production of enough amounts of hydrophobic proteins is considered the most challenging step in making synthetic surfactants. Naturally originated clinical surfactants are limited in amount and may contain pathogenic antigens, so they are difficult to standardize; this means that hydrophobic protein production by the recombinant system is the future to look for. Amounts of recombinant human SP-C have already been produced and used in clinical trials in contrast with recombinant SP-B, which still cannot be produced [31–34]. Table 1 represent available surfactant preparations and their composition.

**Table 1:** Available surfactant preparations and their composition

Preparation	Origin	Protein B	Protein C	Phospholipid	Ref.
**First generation**					
Colfosceril	Synthetic	N/A	N/A	13.5 mg/ml (DPPC)	[30]
Pumactant	Synthetic	N/A	N/A	40 mg/ml (DPPC, phosphatidylglycerol)	[30]
**Animal source**					
Poractant Alfa	Porcine	2–3.7 mg/mM PL	5-11.6 mg/mM PL	80 mg/ml	[30]
Beractant	Bovine	0–1.3 mg/mM PL	1-20 mg/mM PL	25–30 mg/ml	[30]
Calfactant	Calf	5.4 mg/mM PL	8.1 mg/mM PL	35 mg/ml	[30]
SF-RI I	Bovine	2–5.6 mg/mM PL	1-12 mg/mM PL	40 mg/ml	[30]
**Second-generation synthetic surfactant preparations**					
Lucinactant	Synthetic	19.8 mg/mM PL (KL4)	N/A	30 mg/ml (DPPC, POPG)	[30]

Surfactant replacement therapy in treating COVID-19 is an essential case of interest whose results have been promising. Many trials are ongoing in the meantime, indicating improvement of outcomes in patients with COVID-19, as it is known to have antagonism to type 2 pneumocytes whose destruction results in decreased amounts of surfactant and worse prognosis COVID-19 patients [8,35,36].

## Clinical uses of exogenous surfactant

### Exogenous surfactant as a carrier

The concept that surfactants may be used as a delivery vehicle for pulmonary medicines and other chemicals was inspired by the efficacy of exogenous surfactant therapy in neonatal respiratory distress syndrome (NRDS) and the known physicochemical features of surfactants that allow them to diffuse through the lung [37,38]. The fundamental idea is that exogenous surfactant, when coupled, will make it easier to carry a substance to the far reaches of the lung. Surfactant has been employed as a drug carrier in preterm infants at risk of developing bronchopulmonary dysplasia (BPD) [39–41].

### Exogenous surfactant in BPD

BPD, a chronic lung disease, is more likely to develop in premature newborns, especially those who experience prolonged ventilation and high oxygen demands. In two carefully controlled clinical trials, the efficacy of an exogenous surfactant, Survanta®, in combination with budesonide, as compared with the efficacy of the surfactant alone in preventing the development of BPD in preterm newborns [40,42]. A detailed evaluation and meta-analysis of the data from the two clinical trials revealed that BPD incidence was decreased by intra-tracheal injection of budesonide-surfactant mixtures (RR: 0.57; 95%CI: 0.43–0.76, NNT = 5) [43,44]. Before recommending it as routine therapy, additional sizable clinical trials are also necessary due to the lack of rigorous clinical trials. However, these trials offer the first evidence of the theory underlying the clinical application of a surfactant–drug combination and call for further research into the viability of applying this strategy to other medications addressing other clinical diseases like pneumonia and ARDS. Vitamin D has the counterintuitive effect of lessening the severity of COVID-19 based on the up-to-date umbrella review [45], despite the initial inconsistency in the results, which could be explained by genetic polymorphisms in several genes associated with vitamin D metabolisms such as CYP2R1 rs10741657, DHCR7/NADSYN rs12785878, carriers of CYP2R1 GG and DHCR7/NADSYN TG+GG genotypes, and DBP polymorphisms rs4588 and rs7041[46]. Although vitamin D induces the expression of ACE2, which indeed promotes the binding of the virus, it prevents pulmonary vasoconstriction response in COVID-19 cases. Vitamin D may suppress renin activity, lessening angiotensin II production and producing less vasoconstriction [47]. The minimum optimal plasma target of 25-hydroxyvitamin D to be reached in the preventive setting would be ≥30 ng/ml, for which it is necessary to administer high doses of cholecalciferol, also concerning the basal levels of the patient, up to 4000 IU/day [48–50]. Therefore, according to these recommendations, co-administering vitamin D with surfactants may increase its beneficial effect, especially for groups with a higher risk of COVID-19 and vitamin D deficiency.

### Exogenous surfactant in NRDS

NRDS significantly contributed to infant mortality until the mid-1980s, when exogenous surfactant therapy was created [51,52]. It has been demonstrated that intratracheal administration of a pure form of animal surfactants to vulnerable patients significantly increases survival, as reported in network meta-analysis of randomized controlled trials. Hence, the exogenous surfactant remains the preferred therapy in neonatal intensive care units [53,54].

## Clinical efficacy of exogenous pulmonary surfactant

### ARDS

Many studies have been conducted on applying exogenous surfactants in treating ARDS. Cattel et al. studied the clinical efficacy of the exogenous surfactant in the treatment of ARDS. Nine articles supported the exogenous surfactant's clinical effectiveness in inflammatory lung disorders [55]:

- Two are preclinical trials on animals, which explain how surfactant improves lung function and decreases pulmonary edema. The first trial is a comparative study of two pulmonary surfactants, rSP-C33Leu (surfactant protein C analog) and Curosurf® (protectant alfa); improved lung function and reduced inflammation were seen with both surfactant preparations [56]. The second study compares Curosurf® with Synsurf®. Both surfactant preparations ameliorated the oxygenation (significant increase in PaO2/FiO2 ratio) [57].
- Three studies describe clinical trials on infants and children, two of which are meta-analyses [58,59] have demonstrated that pneumothorax, pulmonary interstitial emphysema, and neonatal mortality were all decreased when an exogenous pulmonary surfactant was administered.
  A comparative study compared the administration of pulmonary surfactant in intubated and mechanically ventilated infants (experimental group, *n*=30) and the use of intubation and mechanical ventilation only (control group, *n*=30), and the findings demonstrate that all parameter values improved in both groups, with the experimental group seeing more improvement than the control group [60].
- Two meta-analyses [53,61] describe the results of several randomized clinical studies. The results in the first 24 h following therapy showed that pulmonary surfactant usage increased oxygenation and reduced the duration of ventilation in adults with ARDS or acute lung disease.
- According to two studies [62,63], exogenous surfactants do not improve adult ARDS patients’ oxygenation or mortality while treating inflammatory lung diseases. Another meta-analysis concluded that using exogenous surfactants may improve oxygenation with a pooled mean change of 13.18 mmHg, standard error of 8.23 mmHg; 95% CI: 2.95, 29.32, but not mortality. It was surprising considering the changes in surfactant functions in ARDS patients, and the explanation was that rather than dying from respiratory failure, patients with ARDs typically pass away from the underlying disease’s multi-organ system collapse. Another possibility is that the ideal surfactant recipe has not yet been discovered [64]. It is worth mentioning that ARDS is accompanied by augmented sPLA2 activity in the lungs. sPLA2 degrades the phospholipids of surfactants, including phosphatidylglycerol [11]. In addition to raising surface tension and decreasing lung compliance, a surfactant function deficit may also worsen pulmonary edema [65,66]. It is important to note that the ideal surfactant preparation composition and source may vary depending on whether CARDS or NRDS is being treated. First off, despite the fact that their presence can contribute to a more effective treatment of CARDS, none of the therapeutic surfactants currently available to prevent NRDS contain the hydrophilic proteins SP-A and SP-D. Through the binding of their CRD to the glycosylated spike protein, SP-A and SP-D may aid in the removal of SARS-CoV-2 [24]. It is worth mentioning that potential disease-causing factor, as auto-IgA prevents lung surfactant from reducing surface tension. This might compromise the pulmonary air sacs' ability to stabilize themselves, causing alveolar collapse and inadequate oxygen exchange [67].

### COVID-19

**Figure 4:**
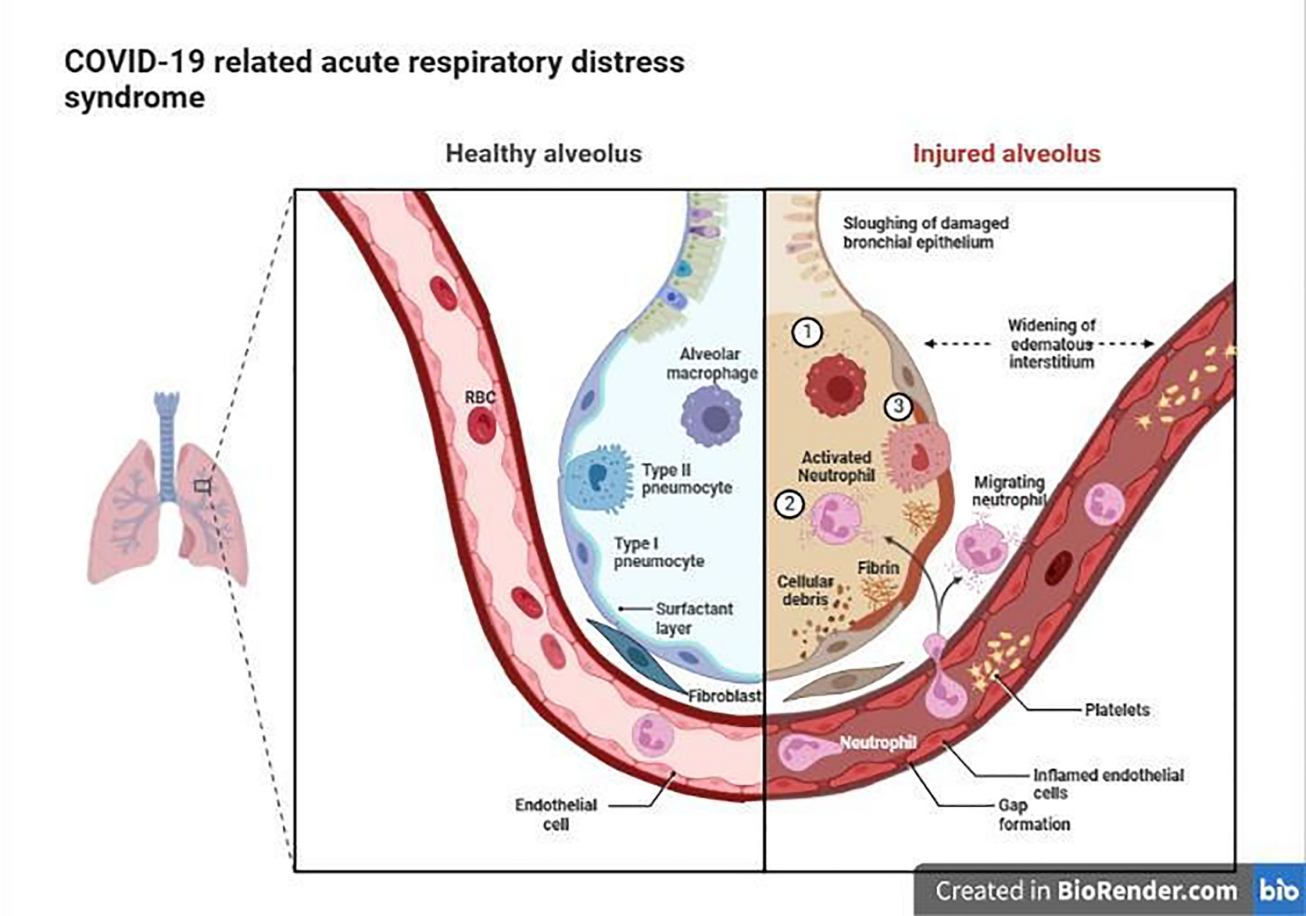
Surfactant replacement therapy showing improved oxygenation and decreased serum leak: I, the restoration of the surfactant layer. II, neutrophil recruitment. III, the neutralization of COVID-19 virus by proteins A and D. IV, recovered surfactant layer

The trials of feasibility and safety of using exogenous pulmonary surfactant showed no direct evidence that surfactant is dysfunctional in the lungs of COVID-19 patients. Type II alveolar cells infected by SARS-CoV-2 could not properly secrete the endogenous surfactant [36,62,63,68,69]. Figure 4 shows the positive effect of surfactant replacement therapy.

Recently, several authors have undertaken ongoing studies to hypothesize exogenous surfactant value in COVID-19 patients [10]. Prospective case–control research [35], one of the most recent investigations on the subject, split the patients into surfactant (*n*=33) and control (*n*=32) groups. A daily dose of 150–300 mg inhaled surfactant was given to the surfactant group. Many clinical outcomes and the oxygenation parameters were examined, and as a result, the surfactant group’s oxygenation index (PaO2/FiO2) significantly increased. Additionally, compared with the control group, considerably fewer patients in the surfactant group were transferred to the intensive care unit and placed in mechanical ventilation. However, mortality did not differ significantly between the groups. Overall, the early findings supported the need for additional research on inhaled surfactant treatment for COVID-19, including randomized controlled clinical trials.

Piva et al. did a retrospective study on seven patients affected by COVID-19 in the case of ARDS compared with 14 individuals who were similar to them in disease characteristics and were given supportive treatment for ARDS simultaneously. Patients with surfactant installed in their small bronchi via bronchoscopy showed no signs of severe decompensation. This study reported a not statistically significant 28-day mortality reduction in the surfactant group. Nevertheless, a favorable safety profile and feasibility of administration without increased risk of infection to medical staff are observed. Future trials may be needed to confirm surfactant efficacy by this route on mortality and morbidity [70].

Infasurf® was given to a COVID-19 patient who was critically unwell at a dosage of 20 mg/kg, according to a report by Heching et al. [8]. A tracheobronchial suction catheter that was inserted through the endotracheal tube was used to administer surfactant into the lungs directly. Following an improvement in oxygenation after 18 h, which continued to improve after 48 h, the patient was taken off from extracorporeal membrane oxygenation and extubated [8]. Busani et al. [9] described giving Curosurf® to five seriously ill patients with pneumonia associated with COVID-19 and poor lung compliance. Soon after the start of invasive mechanical breathing, patients had intratracheal intubation and were administered surfactant for one month at a dose of 30 mg/kg [9]. According to the results, four patients’ oxygen levels improved after an hour, and all patients' oxygen levels improved after 6 h. Also, despite the included patients’ critical condition, a survival rate of 80% over 30 days was seen [9]. Many clinical trials are ongoing on only surfactant supplementation without budesonide or other steroids, e.g., NCT04362059 or NCT04375735 via different drug delivery System [71,72]. However, *in vitro* investigations show that budesonide improves budesonide diffusion over an air–liquid interface without affecting the surfactant's effectiveness [73,74]. Animal studies supported the findings of human studies by showing that budesonide had a longer half-life in the lung than budesonide alone, was more evenly distributed in the alveoli when combined with surfactant than when combined with saline, and reduced lung inflammation without impairing the physiological response to surfactant treatment [75,76]. Table 2 illustrates ongoing trials investigating surfactants’ role in COVID-19 management.

**Table 2:** The ongoing trials that investigate the role of surfactants in COVID-19 management

Preparation	Dose	Dose frequency	Delivery mode	Initiation	Sample size	Ref.
Bovactant (Alveofact®)	1080–3240 mg/kg	3/day	Nebulization	Within 24 h of ventilation	24	[71]
BLES®	50 mg/kg	≤ 3/day	Intratracheal	within 48 h of ventilation	20	[72]
Poractant Alfa (Curosurf®)	48 mg/kg	NA	Endobronchial	Within 72 h of ventilation	20	[77]
Lucinactant (Surfaxin®)	80 mg/kg	NA	Intratracheal	At the time of ventilation	30	[78]
Poractant Alfa	30 mg/kg	3/day	Intratracheal	Within 48 h of ventilation	85	[79]

### Failure of surfactant therapy

Surfactant therapy fails to lower mortality in individuals with ARDS for two primary reasons: First, compared to the edematous, exogenous surfactant-stimulated lungs of preterm newborns, which are known to enhance lung compliance by causing exogenous surfactant to dissolve in water. Even when they are sick, adult lungs still have aerated parts. Because adult lungs typically lack a liquid lining, it is difficult for the surfactant to breakdown in water and ultimately reach the adult lung’s targeted area due to its structural characteristics. In addition, compared to newborn lungs, adult lungs have a more extended airway branching system through which surfactants must travel to reach their intended destinations. Second, the exogenous surfactant loses its surface-active effect even when supplied to the desired disease location. When they travel to the alveoli, the contents of the airways exhibit a range of compositional, physical, chemical, and anomalies in varying degrees. Exogenous surfactants’ surface-active characteristics may change due to their contents [80]. Only the newborn’s need for oxygen therapy is currently used to determine whether surfactant treatment is necessary. Recent recommendations recommend beginning surfactant therapy as soon as possible for patients who need nCPAP of at least 6 cmH_2_O and a FiO_2_ of at least 0.3. Some evidence suggest that lung ultrasound score, as opposed to FiO_2_, better assures prompt surfactant treatment in premature patients, [81,82] and also in early detection of ventilatory associated pneumonia since fluctuations in lung ultrasound scores are correlated with illness severity and progression in adult patients with COVID-19-associated ARDS [83].

### Regulation of surfactant proteins by the proinflammatory cytokines

Activating the proteolytic and oxidative pathways may harm the surfactant complex, resulting in problems including acute respiratory distress syndrome. The collagen-containing C-type lectins or collections include the lectins SP-A and SP-D related to surfactant and the lining of the airways. They act as an opsonin for some bacteria and viruses by aggregating respiratory viruses and microbial binding components [28].

## Surfactant-based prophylaxis against COVID-19

**Figure 5:**
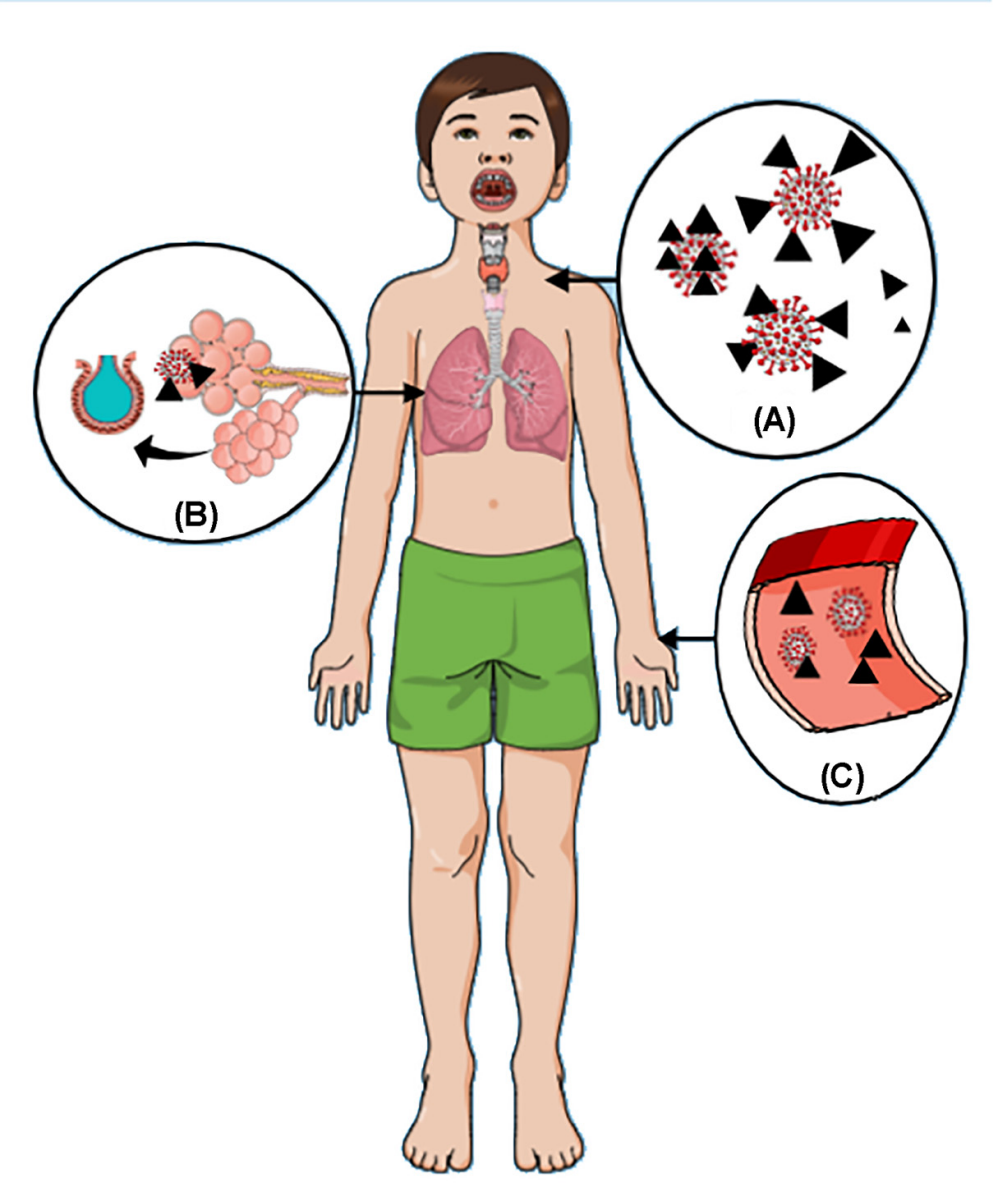
The diagrammatic renderings of the preventative and ( **A**) therapeutic applications of surfactants against COVID-19 The prevention of COVID-19 by a surfactant-based gargle, (**B**) the entry of the SARS-CoV-2 virus into the lung alveoli, and (**C**) the effect of surfactant in circulation The surfactant either covers the virus and makes it inactive or inactivates it.

Coronavirus preferentially attacks type 2 pneumocytes which secrete surfactant resulting in dyspnea and ARDS following viral infection. Surfactant contains several proteins named SP-A, SP-B, SP-C, SP-D, and SP-B stabilizes the lipid coat during respiration. On the other hand, SP-C modulates the compression of lipid coating with decompression during expiration [6]. Scheme of alveolar collapse caused by surfactant impairment due to COVID-19. Pulmonary surfactant also works in host defense against viruses as it works as a barrier to extruding viral invasion by increasing mucociliary transport mechanisms. Also, surfactant components directly interact with respiratory viruses inhibiting their proliferation through stimulating opsonization, inactivation, and agglutination [84,85]. Surfactant phospholipids interact with specific receptors to decrease proinflammatory cytokines, and proteins like SP-A and SP-D act on the host immune response, enhancing viral clearance. Exogenous surfactant showed positive results in treating meconium aspiration syndrome, which has a very similar pathophysiology to COVID-19 as it enhanced oxygenation and its anti-inflammatory effects enhanced wound repairing of damaged alveoli to prevent COVID-19-associated ARDS [64] could be added to SRT. Additionally, SP-A and SP-D are important markers for alveolar collapse which is a very common SARS-CoV-2 infection [86] SARS-CoV-2 enters the body through the lungs via the binding of viral spike protein with ACE-2 receptor. SARS-CoV-2 enters the body primarily through the nose and mouth, with some entrance through the eyes. The virus that entered through these routes can be rendered inactive using a surfactant-based gargle or any other tool filled with surfactant (Figure 5). Gargles and mouthwashes could benefit more if added antiviral drugs, proteases, astringents (protein precipitants). Using surfactants for lung and tracheal infections was not linked to cilia damage. The use of surfactants will stop the virus from attaching via its spike glycoproteins. A virus can be destroyed by interfering with the spike glycoprotein surfactant [15]. Surfactants are considered a physiological barrier to viral infections and function in innate host defense during infections [85]. According to studies, lung surfactant inhalation was linked to a reduction in respiratory illnesses. The antiviral activity is mainly caused by the lipid component [87,88]. Phospholipids found in surfactants play a part in preventing virally induced inflammation and infection [89]. Moreover, the protein component has antiviral properties. By preferential SARS coronavirus spike glycoprotein identification and subsequent activation of macrophages [90,91]. Lately, the use of bear bile to combat COVID-19 has been suggested. Bile salts, which can act as surfactants, are present in bile [92,93].

## The recent promising treatment of COVID-19

Since the first coronavirus epidemic was discovered, no particular and potent antiviral medication or vaccine has been granted approval for the treatment of coronavirus infections. Currently, COVID-19 is mostly treated symptomatically, with supportive care as required. However, a range of therapeutic options are being investigated such as remdesivir and lopinavir, convalescent plasma therapy, phytochemicals, lianhuaqingwen, chloroquine and hydroxychloroquine, and herbal medications [94–96]. Recent studies showed that niclosamide provides distinct advantages over other drugs currently being investigated in the COVID-19 space because it can act as an immunomodulator as well as an anti-bacteriostatic agent [97]. In vitro studies on interferon-α, an antiviral medication routinely used to treat hepatitis, demonstrated that it inhibits SARS-CoV-2 replication. Arbidol, an antiviral medication, has also been reported to have anti-SARS-CoV-2 action [98]. In addition to antiviral drugs, several treatments are being explored [99]. Monoclonal antibodies are being developed as a potential treatment option that can neutralize the virus and prevent it from infecting healthy cells. Immunomodulatory drugs, such as corticosteroids, are also being used to manage severe cases by reducing inflammation and preventing an overactive immune response. Furthermore, vaccines have emerged as a crucial tool in combating COVID-19 [98]. Multiple vaccines have been developed and authorized for emergency use around the world. These vaccines work by stimulating an immune response against SARS-CoV-2, providing protection against infection or reducing its severity if contracted [100].

## Nanoparticle-mediated surfactant therapy in patients with severe COVID-19

Nanotechnology can be used to lessen the negative effects of surfactants and enhance their therapeutic impact. Nanotechnology is used, for example, to enhance the target specificity of surfactant capsules by improving the bioactivities of the encapsulated compounds, reducing side effects, and increasing their solubility and stability. The ability to target surfactants to certain lung areas and extend resistance time in the body are two of the key benefits of employing nanoparticles to distribute surfactants. A wide range of nanomaterials is based on polymer nanoparticles, lipid nanoparticles, inorganic nanoparticles, drug conjugates, and viral nanoparticles [101]. These nanoparticles can have surfactants physically enclosed inside them or covalently coupled to them. An important recent example is that lipid nanoparticles are utilized as mRNA vehicles in BioNTech/Pfizer’s BNT162b2 and Moderna’s mRNA-1273 vaccines. The surfactant-loaded nanoparticles may be injected intravenously. For instance, surfactants can be loaded into poly lactic-glycolic acid nanoparticles or liposomes coated with polyethylene glycol (PEG). The PEG-coated nanoparticles can have longer blood circulation after intravenous injection to delay the rapid clearance of the administered surfactant by macrophages. As a result, surfactants’ negative effects can be reduced while their effectiveness can be increased thanks to nanoparticles [80]. Hydrophilic nanoparticles will more readily bind to SP-A and SP-D, resulting in particle opsonization and improved uptake by AMs and lung dendritic cells [102,103]. Moreover, it is possible to inhale the surfactant-loaded nanoparticles. Though local pulmonary delivery of nanomedicines has many benefits, it can also be hampered by the lung’s numerous extracellular and intracellular barriers, which can prevent siRNA delivery. The destiny of inhaled nanoparticles is generally difficult to predict because it depends on a number of factors. Along with particle-related characteristics like mean mass aerodynamic diameter (MMAD), hydrophobicity, and charge, there are patient-related characteristics like breathing habits and lung anatomy, physiology, and pathology to consider. The highly branching structure of the lungs, where the fate of aerosolized particles after inhalation therapy would significantly rely on their MMAD, is an essential anatomical barrier to pulmonary siRNA delivery [24,104,105]. Surfactants are often delivered to the airways or lung periphery rather than the particular sites of lung injury, which presents a challenge when they are inhaled. According to Dames and colleagues, the surfactant can be loaded in magnetic aerosol droplets to address this issue. Superparamagnetic iron oxide nanoparticles are present in aerosol droplets. A magnetic field can collect aerosol droplets in a particular lung region after inhalation [106]. Additionally, it is anticipated that the side effects of surfactants would be lessened since it interferes less with other organs and tissues. They can therefore be administered both for therapy and as a preventative measure. Bromhexine, on the other hand, blocks transmembrane protease, serine 2 (TMPRSS2). It is thought that inhibiting effective viral entry against SARS-CoV-2 requires TMPRSS2. The first human body-based preliminary exploratory randomized-controlled clinical trial on bromhexine hydrochloride tablets (BHT) to treat COVID-19 was carried out by Wepon Pharmaceutical Group Co. Ltd. based on this supposition. The study’s findings illustrated the advantages of employing BHT from various angles. BHT therapy had few negative side effects while somewhat reducing lung damage. So, clinical testing has shown that bromhexine is effective against COVID-19 [107]. Wu et al. concluded in their study that nanoparticle-medicated surfactant treatment will increase lung compliance and oxygenation by distributing surfactant to targeted lung areas, eventually reducing mortality. Since they can accommodate RNA molecules to increase their stability and improve their intracellular release efficacy, nanoparticles have considerably accelerated the development of RNA therapies over the past few decades [108]. A recent example is the use of lipid nanoparticles as mRNA vehicles in the vaccines BNT162b2 by BioNTech/Pfizer and mRNA-1273 by Moderna [109]. Future clinical trials that assess the effectiveness of nanoparticle-mediated surfactant therapy for patients with COVID-19 ARDS are supported by the success of nanoparticles in the treatment of disease and the development of the COVID-19 vaccine, as well as the positive outcomes of clinical studies of surfactant therapy on COVID-19 patients [80].

Small interfering RNA (siRNA) can be used to address acute respiratory viral infections as it targets both viral and host-related proteins responsible for the infection’s severity and contagion. These siRNA molecules are encapsulated into nanoparticles and need cytosolic delivery to act on their target proteins; however, many intra and extracellular barriers prevent their efficient cytosolic delivery. Depending on the administration technique and lung disease circumstances, exogenous surfactants maintain varying surface-active functions. In order to replace the endogenous surfactant and perform as expected, exogenous surfactants must be administered and delivered to the targeted lung regions with intact or well-preserved surface-active characteristics [80]. This is where the pulmonary surfactant’s role comes in place. Raemdonck and his co-workers stated that exogenous pulmonary surfactant could be used to promote cytosolic siRNA delivery by polymeric nanomedicines [110,111]. The surfactant may be inactivated with a high presence of plasma proteins in airspaces when the blood–gas barrier function is disrupted [106]. There is a need for more effective administration of intact exogenous surfactants with high surface-active characteristics. By delivering surfactant complexes to the areas of the lung that require surfactant the most, nanoparticles have the potential to address these problems, hence reducing ventilator-induced lung injury and mortality.

## Conclusion

For almost four decades, surfactants have been known to treat neonatal respiratory distress syndrome and hyaline membrane disease. Several studies have been conducted to evaluate the use of surfactants in acute respiratory distress syndrome and respiratory distress associated with COVID-19. These studies have demonstrated a positive impact on oxygenation, hospital and intensive care unit stay duration, and the need for mechanical ventilation. However, the limited number of studies, small sample size, unavailability of ongoing trial results, and terminated trials weaken the evidence supporting the standard use of surfactants in adults with COVID-19-associated respiratory distress. Additionally, surfactants have been found to help the innate immune system fight the COVID-19 virus. Surfactants also have the potential to act as liposomal carriers or helpers for certain molecules, drugs, or antivirals to target affected tissue, which presents an excellent area for future research.

## Abbreviations

ACE2: angiotensin-converting enzyme 2
ARDS: acute respiratory distress syndrome
AT2: alveolar type 2
BHT: bromhexine hydrochloride tablet
BPD: bronchopulmonary dysplasia
COVID-19: coronavirus disease 2019
DAMP: damage-associated molecular pattern
DPPC: dipalmitoylphosphatidylcholine
KL4: sinapultide
NRDS: neonatal respiratory distress syndrome
PAMP: pathogen-associated molecular pattern
PEG: polyethylene glycol
POPG: palmitoyloleoyl phosphatidylglycerol
SARS-CoV-2: severe acute respiratory syndrome coronavirus 2
SP: surfactant protien
sPLA2: secretory phospholipase A2
TMPRSS2: transmembrane protease, serine 2

## References

[1] Eva M.A., Janakiram N. and Mauer A. (2021) Left atrial thrombus and covid-19-associated coagulopathy. Chest 160 A153 10.1016/j.chest.2021.07.173

[2] 7Hatmal M.M., Alshaer W., Al-Hatamleh M.A.I., Hatmal M., Smadi O., Taha M.O. et al. (2020) Comprehensive structural and molecular comparison of spike proteins of SARS-CoV-2, SARS-CoV and MERS-CoV, and their interactions with ACE2. Cells 9, 2638 10.3390/cells9122638 33302501 PMC7763676

[3] Cheyne S., Lindley R.I., Smallwood N., Tendal B., Chapman M., Fraile Navarro D. et al. (2022) Care of older people and people requiring palliative care with COVID-19: guidance from the Australian National COVID-19 Clinical Evidence Taskforce. Med. J. Aust. 216, 203–208 10.5694/mja2.51353 34865227 PMC9299653

[4] Mason R.J. (2020) Pathogenesis of COVID-19 from a cell biology perspective. Eur. Respir. J. 55, 2000607 10.1183/13993003.00607-2020 32269085 PMC7144260

[5] Yao Y., Wang H. and Liu Z. (2020) Expression of ACE2 in airways: Implication for COVID-19 risk and disease management in patients with chronic inflammatory respiratory diseases. Clin. Exp. Allergy 50, 1313–1324 10.1111/cea.13746 32975865 PMC7646264

[6] Han S.H. and Mallampalli R.K. (2015) The role of surfactant in lung disease and host defense against pulmonary infections. Ann. Am. Thorac. Soc. 12, 765–774 10.1513/AnnalsATS.201411-507FR 25742123 PMC4418337

[7] Pushpakom S., Iorio F., Eyers P.A., Escott K.J., Hopper S., Wells A. et al. (2018) Drug repurposing: progress, challenges and recommendations. Nat. Rev. Drug Discov. 18, 41–58 10.1038/nrd.2018.168 30310233

[8] Heching M., Lev S., Shitenberg D., Dicker D. and Kramer M.R. (2021) Surfactant for the treatment of ARDS in a patient with COVID-19. Chest 160, e9–e12 10.1016/j.chest.2021.01.028 33493441 PMC7825915

[9] Busani S., Dall'Ara L., Tonelli R., Clini E., Munari E., Venturelli S. et al. (2020) Surfactant replacement might help recovery of low-compliance lung in severe COVID-19 pneumonia. Ther. Adv. Respir. Dis. 14, 10.1177/1753466620951043 32865137 PMC7466887

[10] Seeds M.C., Grier B.L., Suckling B.N., Safta A.M., Long D.L., Waite B.M. et al. (2012) Secretory phospholipase A2-mediated depletion of phosphatidylglycerol in early acute respiratory distress syndrome. Am. J. Med. Sci. 343, 446–451 10.1097/MAJ.0b013e318239c96c 22173044 PMC3307942

[11] Kitsiouli E., Nakos G. and Lekka M.E. (2009) Phospholipase A2 subclasses in acute respiratory distress syndrome. Biochim. Biophys. Acta Mol. Basis Dis. 1792, 941–953 10.1016/j.bbadis.2009.06.007 19577642

[12] Gattinoni L., Chiumello D., Caironi P., Busana M., Romitti F., Brazzi L. et al. (2020) COVID-19 pneumonia: different respiratory treatments for different phenotypes? Intensive Care Med. 46, 1099–1102 10.1007/s00134-020-06033-2 32291463 PMC7154064

[13] Gattinoni L., Coppola S., Cressoni M., Busana M., Rossi S. and Chiumello D. (2020) COVID-19 does not lead to a “typical” acute respiratory distress syndrome. Am. J. Respir. Crit. Care Med. 201, 1299–1300 10.1164/rccm.202003-0817LE 32228035 PMC7233352

[14] De Luca D., Lopez-Rodriguez E., Minucci A., Vendittelli F., Gentile L., Stival E. et al. (2013) Clinical and biological role of secretory phospholipase A2 in acute respiratory distress syndrome infants. Crit. Care 17, R163 10.1186/cc12842 23883784 PMC4057254

[15] Voelker D.R. and Numata M. (2019) Phospholipid regulation of innate immunity and respiratory viral infection. J. Biol. Chem. 294, 4282–4289 10.1074/jbc.AW118.003229 30733339 PMC6433062

[16] Fleming B.D. and Keough K.M.W. (1988) Surface respreading after collapse of monolayers containing major lipids of pulmonary surfactant. Chem. Phys. Lipids. 49, 81–86 10.1016/0009-3084(88)90067-9 3233714

[17] Bernhard W. (2016) Lung surfactant: Function and composition in the context of development and respiratory physiology. Ann. Anat. 208, 146–150 10.1016/j.aanat.2016.08.003 27693601

[18] Hallman M., Kulovich M., Kirkpatrick E., Sugarman R.G. and Gluck L. (1976) Phosphatidylinositol and phosphatidylglycerol in amniotic fluid: indices of lung maturity. Am. J. Obstet. Gynecol. 125, 613–617 10.1016/0002-9378(76)90782-1 180804

[19] Castillo-Sánchez J.C., Cruz A. and Pérez-Gil J. (2021) Structural hallmarks of lung surfactant: lipid-protein interactions, membrane structure and future challenges. Arch. Biochem. Biophys. 703, 108850 10.1016/j.abb.2021.108850 33753033

[20] Cañadas O., Olmeda B., Alonso A. and Pérez-Gil J. (2020) Lipid-protein and protein-protein interactions in the pulmonary surfactant system and their role in lung homeostasis. Int. J. Mol. Sci. 21, 3708 10.3390/ijms21103708 32466119 PMC7279303

[21] Pérez-Gil J. (2008) Structure of pulmonary surfactant membranes and films: the role of proteins and lipid-protein interactions. Biochim. Biophys. Acta Biomembr. 1778, 1676–1695 10.1016/j.bbamem.2008.05.003 18515069

[22] Arroyo R., Khan M.A., Echaide M., Pérez-Gil J. and Palaniyar N. (2019) SP-D attenuates LPS-induced formation of human neutrophil extracellular traps (NETs), protecting pulmonary surfactant inactivation by NETs. Commun. Biol. 2, 1–13 10.1038/s42003-019-0662-5 31872075 PMC6915734

[23] Feingold K.R. and Grunfeld C. (2000) The effect of inflammation and infection on lipids and lipoproteins MDText.com, Inc. http://www.ncbi.nlm.nih.gov/books/NBK326741/South Dartmouth (MA)

[24] Herman L., De Smedt S.C. and Raemdonck K. (2022) Pulmonary surfactant as a versatile biomaterial to fight COVID-19. J. Control. Release 342, 170–188 10.1016/j.jconrel.2021.11.023 34813878 PMC8605818

[25] Blanco O. and Pérez-Gil J. (2007) Biochemical and pharmacological differences between preparations of exogenous natural surfactant used to treat Respiratory Distress Syndrome: Role of the different components in an efficient pulmonary surfactant. Eur. J. Pharmacol. 568, 1–15 10.1016/j.ejphar.2007.04.035 17543939

[26] Liekkinen J., Enkavi G., Javanainen M., Olmeda B., Pérez-Gil J. and Vattulainen I. (2020) Pulmonary surfactant lipid reorganization induced by the adsorption of the oligomeric surfactant protein B complex. J. Mol. Biol. 432, 3251–3268 10.1016/j.jmb.2020.02.028 32135191

[27] Taeusch H.W., De La Serna J.B., Perez-Gil J., Alonso C. and Zasadzinski J.A. (2005) Inactivation of pulmonary surfactant due to serum-inhibited adsorption and reversal by hydrophilic polymers: Experimental. Biophys. J. 89, 1769–1779 10.1529/biophysj.105.062620 15923228 PMC1366680

[28] Jeon G.W., Oh M. and Sin J.B. (2015) Efficacy of surfactant-TA, calfactant and poractant alfa for preterm infants with respiratory distress syndrome: A retrospective study. Yonsei Med. J. 56, 433–439 10.3349/ymj.2015.56.2.433 25683992 PMC4329355

[29] Ramanathan R. (2009) Animal-derived surfactants: Where are we? The evidence from randomized, controlled clinical trials J. Perinatol. 29, S38–S43 10.1038/jp.2009.31 19399008

[30] Logan J.W. and Moya F.R. (2009) Animal-derived surfactants for the treatment and prevention of neonatal respiratory distress syndrome: Summary of clinical trials. Ther. Clin. Risk Manag. 5, 251–260 10.2147/tcrm.s4029 19436610 PMC2697515

[31] Hafner D., Germann P.G. and Hauschke D. (1998) Effects of rSP-C surfactant on oxygenation and histology in a rat-lung- lavage model of acute lung injury. Am. J. Respir. Crit. Care Med. 158, 270–278 10.1164/ajrccm.158.1.9712061 9655740

[32] Lukovic D., Plasencia I., Taberner F.J., Salgado J., Calvete J.J., Pérez-Gil J. et al. (2006) Production and characterisation of recombinant forms of human pulmonary surfactant protein C (SP-C): Structure and surface activity. Biochim. Biophys. Acta Biomembr. 1758, 509–518 10.1016/j.bbamem.2006.03.005 16631109

[33] Spragg R.G., Taut F.J.H., Lewis J.F., Schenk P., Ruppert C., Dean N. et al. (2011) Recombinant surfactant protein C-based surfactant for patients with severe direct lung injury. Am. J. Respir. Crit. Care Med. 183, 1055–1061 10.1164/rccm.201009-1424OC 21148720

[34] Bañares-Hidalgo Á., Pérez-Gil J. and Estrada P. (2016) Conformational stability of the NH2-terminal propeptide of the precursor of pulmonary surfactant protein SP-B. PLoS ONE 11, e0158430 10.1371/journal.pone.0158430 27380171 PMC4933373

[35] Avdeev S.N., Trushenko N.V., Chikina S.Y., Tsareva N.A., Merzhoeva Z.M., Yaroshetskiy A.I. et al. (2021) Beneficial effects of inhaled surfactant in patients with COVID-19-associated acute respiratory distress syndrome. Respir. Med. 185, 106489 10.1016/j.rmed.2021.106489 34087610 PMC8163691

[36] Veldhuizen R.A.W., Zuo Y.Y., Petersen N.O., Lewis J.F. and Possmayer F. (2021) The COVID-19 pandemic: a target for surfactant therapy? Expert Rev. Respir. Med. 15, 597–608 10.1080/17476348.2021.1865809 33331197

[37] Morales J.O., Peters J.I. and Williams R.O. (2011) Surfactants: their critical role in enhancing drug delivery to the lungs. Ther. Deliv. 2, 623–641 10.4155/tde.11.15 22833979

[38] Hidalgo A., Cruz A. and Pérez-Gil J. (2015) Barrier or carrier? Pulmonary surfactant and drug delivery Eur. J. Pharm. Biopharm. 95, 117–127 10.1016/j.ejpb.2015.02.014 25709061

[39] Abdel-Latif M.E., Davis P.G., Wheeler K.I., De Paoli A.G. and Dargaville P.A. (2021) Surfactant therapy via thin catheter in preterm infants with or at risk of respiratory distress syndrome. Cochrane Database Systematic Rev. 2021 CD011672 10.1002/14651858.CD011672.pub2 PMC8109227 33970483

[40] Boel L., Banerjee S. and Chakraborty M. (2018) Postnatal steroids in extreme preterm infants: Intra-tracheal instillation using surfactant as a vehicle. Paediatr. Respir. Rev. 25, 78–84 10.1016/j.prrv.2017.05.002 28651937

[41] Roberts K., Stepanovich G., Bhatt-Mehta V. and Donn S.M. (2021) New pharmacologic approaches to bronchopulmonary dysplasia. J. Exp. Pharmacol. 13, 377–396 10.2147/JEP.S262350 33790663 PMC8006962

[42] Lin C.H., Jeng M.J., Kuo B.I.T. and Kou Y.R. (2016) Effects of surfactant lavage combined with intratracheal budesonide instillation on meconium-injured piglet lungs. Pediatr. Crit. Care Med. 17, e287–295 10.1097/PCC.0000000000000729 27124561

[43] Venkataraman R., Kamaluddeen M., Hasan S.U., Robertson H.L. and Lodha A. (2017) Intratracheal administration of budesonide-surfactant in prevention of bronchopulmonary dysplasia in very low birth weight infants: a systematic review and meta-analysis. Pediatr. Pulmonol. 52, 968–975 10.1002/ppul.23680 28165675

[44] Hascoët J.M., Picaud J.C., Ligi I., Blanc T., Daoud P., Zupan V. et al. (2018) Review shows that using surfactant a number of times or as a vehicle for budesonide may reduce the risk of bronchopulmonary dysplasia. Acta Paediatrica Int. J. Paediatrics 107, 1140–1144 10.1111/apa.14171 29193276

[45] Petrelli F., Oldani S., Borgonovo K., Cabiddu M., Dognini G., Ghilardi M. et al. (2023) Vitamin D3 and COVID-19 outcomes: an umbrella review of systematic reviews and meta-analyses. Antioxidants 12, 247 10.3390/antiox12020247 36829806 PMC9952713

[46] Abouzid M., Główka F., Kagan L. and Karaźniewicz-Łada M. (2022) Vitamin D metabolism gene polymorphisms and their associated disorders: a literature review. Curr. Drug Metab. 23, 630–651 10.2174/1389200223666220627104139 35761493

[47] Kumar D., Gupta P. and Banerjee D. (2020) Letter: does vitamin D have a potential role against COVID-19? Aliment. Pharmacol. Ther. 52, 409–411 10.1111/apt.15801 32432810 PMC7276741

[48] Balla M., Merugu G.P., Konala V.M., Sangani V., Kondakindi H., Pokal M. et al. (2020) Back to basics: review on vitamin D and respiratory viral infections including COVID-19. J. Community Hosp. Intern. Med. Perspect. 10, 529–536 10.1080/20009666.2020.1811074 33194123 PMC7599018

[49] Arboleda J.F. and Urcuqui-Inchima S. (2020) Vitamin D supplementation: a potential approach for coronavirus/COVID-19 therapeutics? Front. Immunol. 11, 1523 10.3389/fimmu.2020.01523 32655583 PMC7324720

[50] Maghbooli Z., Sahraian M.A., Ebrahimi M., Pazoki M., Kafan S., Tabriz H.M. et al. (2020) Vitamin D sufficiency, a serum 25-hydroxyvitamin D at least 30 ng/mL reduced risk for adverse clinical outcomes in patients with COVID-19 infection. PLoS ONE 15, e0239799 10.1371/journal.pone.0239799 32976513 PMC7518605

[51] Scott B.L., Bonadonna D., Ozment C.P. and Rehder K.J. (2021) Extracorporeal membrane oxygenation in critically ill neonatal and pediatric patients with acute respiratory failure: a guide for the clinician. Expert Rev. Respir. Med. 15, 1281–1291 10.1080/17476348.2021.1932469 34010072

[52] Halliday H.L. (2008) Surfactants: Past, present and future. J. Perinatol. 28, S47–S56 10.1038/jp.2008.50 18446178 PMC7104445

[53] Zhang L., Cao H.Y., Zhao S., Yuan L.J., Han D., Jiang H. et al. (2015) Effect of exogenous pulmonary surfactants on mortality rate in neonatal respiratory distress syndrome: A network meta-analysis of randomized controlled trials. Pulm. Pharmacol. Ther. 34, 46–54 10.1016/j.pupt.2015.08.005 26296793

[54] De Luca D., Cogo P., Kneyber M.C., Biban P., Semple M.G., Perez-Gil J. et al. (2021) Surfactant therapies for pediatric and neonatal ARDS: ESPNIC expert consensus opinion for future research steps. Crit. Care 25, 75 10.1186/s13054-021-03489-6 33618742 PMC7898495

[55] Cattel F., Giordano S., Bertiond C., Lupia T., Corcione S., Scaldaferri M. et al. (2021) Use of exogenous pulmonary surfactant in acute respiratory distress syndrome (ARDS): Role in SARS-CoV-2-related lung injury. Respir. Physiol. Neurobiol. 288, 103645 10.1016/j.resp.2021.103645 33657448 PMC7916525

[56] Zebialowicz Ahlström J., Massaro F., Mikolka P., Feinstein R., Perchiazzi G., Basabe-Burgos O. et al. (2019) Synthetic surfactant with a recombinant surfactant protein C analogue improves lung function and attenuates inflammation in a model of acute respiratory distress syndrome in adult rabbits. Respir. Res. 20, 245 10.1186/s12931-019-1220-x 31694668 PMC6836435

[57] van Zyl J.M. and Smith J. (2013) Surfactant treatment before first breath for respiratory distress syndrome in preterm lambs: Comparison of a peptide-containing synthetic lung surfactant with porcine-derived surfactant. Drug Des. Devel. Ther. 7, 905–916 10.2147/DDDT.S47270 PMC3769412 24039400

[58] Chakraborty M. and Kotecha S. (2013) Pulmonary surfactant in newborn infants and children. Breathe 9, 476–488 10.1183/20734735.006513

[59] More K., Sakhuja P. and Shah P.S. (2014) Minimally invasive surfactant administration in preterm infants: A meta-narrative review. JAMA Pediatr. 168, 901–908 10.1001/jamapediatrics.2014.1148 25089718

[60] Liu J., Liu G., Wu H. and Li Z. (2017) Efficacy study of pulmonary surfactant combined with assisted ventilation for acute respiratory distress syndrome management of term neonates. Exp. Ther. Med. 14, 2608–2612 10.3892/etm.2017.4839 28947918 PMC5609315

[61] Meng H., Sun Y., Lu J., Fu S., Meng Z., Scott M. et al. (2012) Exogenous surfactant may improve oxygenation but not mortality in adult patients with acute lung injury/acute respiratory distress syndrome: A meta-analysis of 9 clinical trials. J. Cardiothorac. Vasc. Anesth. 26, 849–856 10.1053/j.jvca.2011.11.006 22265270 PMC9942513

[62] Dushianthan A., Cusack R., Goss V., Postle A.D. and Grocott M.P.W. (2012) Clinical review: Exogenous surfactant therapy for acute lung injury/acute respiratory distress syndrome - where do we go from here? Crit. Care 16, 238 10.1186/cc11512 23171712 PMC3672556

[63] Lorenzo B. (2020) Covid-19, type II alveolar cells and surfactant. J. Med. Clin. Res. Rev. 4, 1–3 10.33425/2639-944X.1136

[64] Davidson W.J., Dorscheid D., Spragg R., Schulzer M., Mak E. and Ayas N.T. (2006) Exogenous pulmonary surfactant for the treatment of adult patients with acute respiratory distress syndrome: Results of a meta-analysis. Crit. Care 10, R41 10.1186/cc4851 16542488 PMC1550886

[65] Nugent K., Dobbe L., Rahman R., Elmassry M. and Paz P. (2019) Lung morphology and surfactant function in cardiogenic pulmonary edema: A narrative review. J. Thorac. Dis. 11, 4031–4038 10.21037/jtd.2019.09.02 31656679 PMC6790471

[66] Albert R.K., Lakshminarayan S., Hildebrandt J., Kirk W. and Butler J. (1979) Increased surface tension favors pulmonary edema formation in anesthetized dogs' lungs. J. Clin. Invest. 63, 1015–1018 10.1172/JCI109369 447823 PMC372043

[67] Sinnberg T., Lichtensteiger C., Ali O.H., Pop O.T., Jochum A.K., Risch L. et al. (2023) Pulmonary surfactant proteins are inhibited by immunoglobulin A autoantibodies in severe COVID-19. Am. J. Respir. Crit. Care Med. 207, 38–49 10.1164/rccm.202201-0011OC 35926164 PMC9952873

[68] Raghavendran K., Willson D. and Notter R.H. (2011) Surfactant therapy for acute lung injury and acute respiratory distress syndrome. Crit. Care Clin. 27, 525–559 10.1016/j.ccc.2011.04.005 21742216 PMC3153076

[69] Bollag W.B. and Gonzales J.N. (2020) Phosphatidylglycerol and surfactant: A potential treatment for COVID-19? Med. Hypotheses 144, 110277 10.1016/j.mehy.2020.110277 33254581 PMC7493731

[70] Piva S., DiBlasi R.M., Slee A.E., Jobe A.H., Roccaro A.M., Filippini M. et al. (2021) Surfactant therapy for COVID-19 related ARDS: a retrospective case-control pilot study. Respir. Res. 22, 20 10.1186/s12931-020-01603-w 33461535 PMC7812332

[71] NCT04362059 (2020) A clinical trial of nebulized surfactant for the treatment of moderate to severe COVID-19. https://classic.clinicaltrials.gov/ct2/show/NCT04362059 Accessed 2023-01-24

[72] NCT04375735 (2020) London's exogenous surfactant study for COVID19. https://classic.clinicaltrials.gov/ct2/show/NCT04375735, Accessed 2022-09-25

[73] Ricci F., Catozzi C., Ravanetti F., Murgia X., D'Aló F., Macchidani N. et al. (2017) In vitro and in vivo characterization of poractant alfa supplemented with budesonide for safe and effective intratracheal administration. Pediatr. Res. 82, 1056–1063 10.1038/pr.2017.171 28723887

[74] Zhang H., Wang Y.E., Neal C.R. and Zuo Y.Y. (2012) Differential effects of cholesterol and budesonide on biophysical properties of clinical surfactant. Pediatr. Res. 71, 316–323 10.1038/pr.2011.78 22391630 PMC3338335

[75] Hidalgo A., Salomone F., Fresno N., Orellana G., Cruz A. and Perez-Gil J. (2017) Efficient interfacially driven vehiculization of corticosteroids by pulmonary surfactant. Langmuir 33, 7929–7939 10.1021/acs.langmuir.7b01177 28738158

[76] Yeh T.F., Chen C.M., Wu S.Y., Husan Z., Li T.C., Hsieh W.S. et al. (2016) Intratracheal administration of budesonide/surfactant to prevent bronchopulmonary dysplasia. Am. J. Respir. Crit. Care Med. 193, 86–95 10.1164/rccm.201505-0861OC 26351971

[77] Nct (2020) Curosurf® in Adult Acute Respiratory Distress Syndrome Due to COVID-19. https://classic.clinicaltrials.gov/ct2/show/NCT04384731 Accessed 2023-02-15

[78] Sabater-Cruz N., Martinez-Conesa E., Vilarrodona A. and Casaroli-Marano R.P. (2022) Lyophilized amniotic membrane graft for primary pterygium surgery: preliminary results. Cell Tissue Bank. 23, 401–406 10.1007/s10561-021-09955-3 34628551

[79] NCT04502433 (2020) Poractant Alfa - Curosurf and SARS-COV-19 ARDS (Covid-19). https://classic.clinicaltrials.gov/ct2/show/NCT04502433 Accessed 2022-10-04

[80] Wu Y., Li X., Gan Y. and Zhao C. (2021) Nanoparticle-mediated surfactant therapy in patients with severe COVID-19: a perspective. J. Mater. Chem. B. 9, 6988–6993 10.1039/D1TB00730K 34085075

[81] Sweet D.G., Carnielli V., Greisen G., Hallman M., Ozek E., Te Pas A. et al. (2019) European consensus guidelines on the management of respiratory distress syndrome - 2019 Update. Neonatology 115, 432–450 10.1159/000499361 30974433 PMC6604659

[82] Wright C.J., Glaser K., Speer C.P., Härtel C. and Roehr C.C. (2023) Response to the Letter to the Editor “Lung ultrasound score is the forgotten, accurate and physiologically sound method to guide surfactant administration” by Raimondi et al., 2022. J. Pediatr. 257 113363 10.1016/j.jpeds.2023.02.009 36828345

[83] Dargent A., Chatelain E., Si-Mohamed S., Simon M., Baudry T., Kreitmann L. et al. (2021) Lung ultrasound score as a tool to monitor disease progression and detect ventilator-associated pneumonia during COVID-19-associated ARDS. Heart Lung 50, 700–705 10.1016/j.hrtlng.2021.05.003 34107394 PMC8165084

[84] Zuo Y.Y., Veldhuizen R.A.W., Neumann A.W., Petersen N.O. and Possmayer F. (2008) Current perspectives in pulmonary surfactant - Inhibition, enhancement and evaluation. Biochim. Biophys. Acta Biomembr. 1778, 1947–1977 10.1016/j.bbamem.2008.03.021 18433715

[85] Hsieh I.N., De Luna X., White M.R. and Hartshorn K.L. (2018) The role and molecular mechanism of action of surfactant protein D in innate host defense against influenza A virus. Front. Immunol. 9, 10.3389/fimmu.2018.01368 PMC6008380 29951070

[86] Shi H., Han X., Jiang N., Cao Y., Alwalid O., Gu J. et al. (2020) Radiological findings from 81 patients with COVID-19 pneumonia in Wuhan, China: a descriptive study. Lancet Infect. Dis. 20, 425–434 10.1016/S1473-3099(20)30086-4 32105637 PMC7159053

[87] Glasser J.R. and Mallampalli R.K. (2012) Surfactant and its role in the pathobiology of pulmonary infection. Microbes Infect. 14, 17–25 10.1016/j.micinf.2011.08.019 21945366 PMC3247641

[88] Perino J., Crouzier D., Spehner D., Debouzy J.C., Garin D., Crance J.M. et al. (2011) Lung surfactant DPPG phospholipid inhibits vaccinia virus infection. Antiviral Res. 89, 89–97 10.1016/j.antiviral.2010.11.009 21095206

[89] Numata M., Mitchell J.R., Tipper J.L., Brand J.D., Trombley J.E., Nagashima Y. et al. (2020) Pulmonary surfactant lipids inhibit infections with the pandemic H1N1 influenza virus in several animal models. J. Biol. Chem. 295, 1704–1715 10.1074/jbc.RA119.012053 31882535 PMC7008372

[90] van Eijk M., Hillaire M.L.B., Rimmelzwaan G.F., Rynkiewicz M.J., White M.R., Hartshorn K.L. et al. (2019) Enhanced antiviral activity of human surfactant protein d by site-specific engineering of the carbohydrate recognition domain. Front. Immunol. 10, 10.3389/fimmu.2019.02476 PMC6842947 31749796

[91] Leth-Larsen R., Zhong F., Chow V.T.K., Holmskov U. and Lu J. (2007) The SARS coronavirus spike glycoprotein is selectively recognized by lung surfactant protein D and activates macrophages. Immunobiology 212, 201–211 10.1016/j.imbio.2006.12.001 17412287 PMC7114820

[92] Tadros T. (2014) Chapter 2 - Colloid and interface aspects of pharmaceutical science. Colloid and Interface Science in Pharmaceutical Research and Development 29–54 Elsevier, Amsterdam, 978-0-444-62614-1 10.1016/B978-0-444-62614-1.00002-8

[93] Subramanian S., Iles T., Ikramuddin S. and Steer C.J. (2020) Merit of an ursodeoxycholic acid clinical trial in COVID-19 patients. Vaccines (Basel) 8, 320 10.3390/vaccines8020320 32575350 PMC7350268

[94] Rai P.K., Mueed Z., Chowdhury A., Deval R., Kumar D., Kamal M.A. et al. (2021) Current Overviews on COVID-19 Management Strategies. Curr. Pharm. Biotechnol. 23 361–387 10.2174/1389201022666210509022313 33966618

[95] Gurwitz D. (2020) Angiotensin receptor blockers as tentative SARS-CoV-2 therapeutics. Drug Dev. Res. 81, 537–540 10.1002/ddr.21656 32129518 PMC7228359

[96] Walls A.C., Park Y.J., Tortorici M.A., Wall A., McGuire A.T. and Veesler D. (2020) Structure, Function, and Antigenicity of the SARS-CoV-2 Spike Glycoprotein. Cell 181, 281–292.e6 10.1016/j.cell.2020.02.058 32155444 PMC7102599

[97] Singh S., Weiss A., Goodman J., Fisk M., Kulkarni S., Lu I. et al. (2022) Niclosamide—A promising treatment for COVID-19. Br. J. Pharmacol. 179, 3250–3267 10.1111/bph.15843 35348204 PMC9111792

[98] Wijayasinghe Y.S., Bhansali P., Viola R.E., Kamal M.A. and Poddar N.K. (2020) Natural Products: A Rich Source of Antiviral Drug Lead Candidates for the Management of COVID-19. Curr. Pharm. Des. 27 3526–3550 10.2174/1381612826666201118111151 33213322

[99] Zhu Z., Lu Z., Xu T., Chen C., Yang G., Zha T. et al. (2020) Arbidol monotherapy is superior to lopinavir/ritonavir in treating COVID-19. J. Infect. 81, e21–e23 10.1016/j.jinf.2020.03.060 32283143 PMC7195393

[100] Ullah I., Khan K.S., Tahir M.J., Ahmed A. and Harapan H. (2021) Myths and conspiracy theories on vaccines and COVID-19: Potential effect on global vaccine refusals. Vacunas 22, 93–97 10.1016/j.vacun.2021.01.001 33727904 PMC7951562

[101] Aghebati-Maleki A., Dolati S., Ahmadi M., Baghbanzhadeh A., Asadi M., Fotouhi A. et al. (2020) Nanoparticles and cancer therapy: Perspectives for application of nanoparticles in the treatment of cancers. J. Cell. Physiol. 235, 1962–1972 10.1002/jcp.29126 31441032

[102] Ruge C.A., Kirch J., Cañadas O., Schneider M., Perez-Gil J., Schaefer U.F. et al. (2011) Uptake of nanoparticles by alveolar macrophages is triggered by surfactant protein A. Nanomedicine 7, 690–693 10.1016/j.nano.2011.07.009 21839052

[103] Ruge C., Schäfer U., Kirch J., Schneider M., Cañadas O., Perez-Gil J. et al. (2011) Role of Surfactant Proteins in the interaction of nanoparticles with the air-blood-barrier. Pneumologie 65, A50 10.1055/s-0031-1296141

[104] Hu G., Jiao B., Shi X., Valle R.P., Fan Q. and Zuo Y.Y. (2013) Physicochemical properties of nanoparticles regulate translocation across pulmonary surfactant monolayer and formation of lipoprotein corona. ACS Nano 7, 10525–10533 10.1021/nn4054683 24266809 PMC5362675

[105] Raesch S.S., Tenzer S., Storck W., Rurainski A., Selzer D., Ruge C.A. et al. (2015) Proteomic and Lipidomic Analysis of Nanoparticle Corona upon Contact with Lung Surfactant Reveals Differences in Protein, but Not Lipid Composition. ACS Nano 9, 11872–11885 10.1021/acsnano.5b04215 26575243

[106] Dames P., Gleich B., Flemmer A., Hajek K., Seidl N., Wiekhorst F. et al. (2007) Targeted delivery of magnetic aerosol droplets to the lung. Nat. Nanotechnol. 2, 495–499 10.1038/nnano.2007.217 18654347

[107] Depfenhart M., de Villiers D., Lemperle G., Meyer M. and Di Somma S. (2020) Potential new treatment strategies for COVID-19: is there a role for bromhexine as add-on therapy? Intern. Emerg. Med. 15, 801–812 10.1007/s11739-020-02383-3 32458206 PMC7249615

[108] Tao W., Yurdagul A., Kong N., Li W., Wang X., Doran A.C. et al. (2020) SiRNA nanoparticles targeting CaMKIIγ in lesional macrophages improve atherosclerotic plaque stability in mice. Sci. Transl. Med. 12, 10.1126/scitranslmed.aay1063 PMC7476570 32718990

[109] Cheng P. and Pu K. (2021) Molecular imaging and disease theranostics with renal-clearable optical agents. Nat. Rev. Mater. 6, 1095–1113 10.1038/s41578-021-00328-6

[110] Guagliardo R., Herman L., Penders J., Zamborlin A., De Keersmaecker H., Van De Vyver T. et al. (2021) Surfactant protein b promotes cytosolic sirna delivery by adopting a virus-like mechanism of action. ACS Nano 15, 8095–8109 10.1021/acsnano.0c04489 33724778

[111] Uludağ H., Parent K., Aliabadi H.M. and Haddadi A. (2020) Prospects for RNAi Therapy of COVID-19. Front. Bioeng. Biotechnol. 8, 10.3389/fbioe.2020.00916 PMC7409875 32850752

